# Damage to Orbitofrontal Areas 12 and 13, but Not Area 14, Results in Blunted Attention and Arousal to Socioemotional Stimuli in Rhesus Macaques

**DOI:** 10.3389/fnbeh.2020.00150

**Published:** 2020-09-08

**Authors:** Lauren E. Murphy, Jocelyne Bachevalier

**Affiliations:** ^1^Department of Psychology, Emory College of Arts and Sciences, Emory University, Atlanta, GA, United States; ^2^Yerkes National Primate Research Center, Emory University, Atlanta, GA, United States

**Keywords:** orbitofrontal cortex (OFC), emotion, *Macaca mulatta*, face processing, social processing, eye-tracking, pupil dilation

## Abstract

An earlier study in monkeys indicated that lesions to the mid-portion of the ventral orbitofrontal cortex (OFC), including Walker’s areas 11 and 13 (OFC11/13), altered the spontaneous scanning of still pictures of primate faces (neutral and emotional) and the modulation of arousal. Yet, these conclusions were limited by several shortcomings, including the lesion approach, use of static rather than dynamic stimuli, and manual data analyses. To confirm and extend these earlier findings, we compared attention and arousal to social and nonsocial scenes in three groups of rhesus macaques with restricted lesions to one of three OFC areas (OFC12, OFC13, or OFC14) and a sham-operated control group using eye-tracking to capture scanning patterns, focal attention and pupil size. Animals with damage to the lateral OFC areas (OFC12 and OFC13) showed decreased attention specifically to the eyes of negative (threatening) social stimuli and increased arousal (increased pupil diameter) to positive social scenes. In contrast, animals with damage to the ventromedial OFC area (OFC14) displayed no differences in attention or arousal in the presence of social stimuli compared to controls. These findings support the notion that areas of the lateral OFC are critical for directing attention and modulating arousal to emotional social cues. Together with the existence of face-selective neurons in these lateral OFC areas, the data suggest that the lateral OFC may set the stage for multidimensional information processing related to faces and emotion and may be involved in social judgments.

## Introduction

The ability to flexibly control and update emotional response to a stimulus, known as emotion regulation, is critical for survival in many species, particularly in group-living, social mammals. The orbitofrontal cortex (OFC) has been suggested as one of the critical brain areas supporting this function as damage to this cortical area in humans and nonhuman primates results in blunted responses to emotionally relevant cues (Saver and Damasio, 1991; Bechara et al., 1997; Blair and Cipolotti, 2000; Beer et al., 2003; Izquierdo et al., 2005; Kalin et al., 2007; Machado and Bachevalier, 2008; Noonan et al., 2010; Kazama et al., 2014; Rudebeck et al., 2013) and in an inability to flexibly modulate emotional responses in accordance with changing contexts or values (Butter, 1969; Jones and Mishkin, 1972; Rolls et al., 1994; Dias et al., 1996; O’Doherty et al., 2001; Fellows and Farah, 2003; Hornak et al., 2004; Wallis, 2007; Noonan et al., 2011; Rudebeck and Murray, 2011; Walton et al., 2011; Kazama et al., 2014; Murray et al., 2015). Whether these behavioral alterations result from disruption of separable functions mediated by different subfields of the OFC remains an outstanding question that is gaining increased attention.

The primate OFC is a heteromodal association area that receives converging inputs from multiple sensory systems, including visual (Pandya and Kuypers, 1969; Barbas, 1988, 1993, 1995; Barbas and Pandya, 1989; Seltzer and Pandya, 1989; Morecraft et al., 1992; Webster et al., 1994; Carmichael and Price, 1995) and auditory inputs (Barbas, 1988, 1993; Hackett et al., 1998; Romanski et al., 1999; Romanski and Goldman-Rakic, 2002) as well as inputs from subcortical structures, such as the amygdala and hypothalamus, critical for emotion regulation and arousal (Barbas and De Olmos, 1990; Carmichael and Price, 1995; Rempel-Clower and Barbas, 1998; Barbas et al., 2003; Ghashghaei et al., 2007). The OFC is composed of several cytoarchitectonic subfields that have different connectional networks. Based on their connectional networks, the OFC subfields appear to comprise two main networks supporting different and complementary functions. In monkeys, the lateral OFC network (lOFC) is composed of Walker’s area 12 on the ventrolateral border of OFC as well as Walker’s area 13 in the mid-portion OFC between the lateral and orbital sulci. The lateral OFC network receives robust inputs from all sensory modalities and is reciprocally connected with the amygdala (Carmichael and Price, 1995, 1996; Price, 2006), suggesting that this part of the OFC may be specialized for the assessment and valuation of sensory cues from objects, food, faces and voices (Blair et al., 1999; Gorno-Tempini et al., 2001; Mitchell et al., 2003; Watson and Platt, 2012). By contrast, the medial OFC network includes the more ventromedial portion of the OFC (vmOFC), which encompasses Walker’s area 14 and other areas (areas 25, 32 and anterior cingulate areas) on the medial aspect of the hemisphere. The vmOFC receives fewer inputs from sensory cortical areas, and is instead densely interconnected with structures that modulate autonomic arousal (amygdala and hypothalamus) and with other parts of the medial frontal cortex, such as the anterior cingulate areas (Carmichael and Price, 1995; Rempel-Clower and Barbas, 1998). The differences in anatomical connectivity patterns between the lOFC and vmOFC have prompted recent studies into the functional distinctions between these two OFC networks, suggesting distinct contributions to aspects of reward-guided behavior and value-based decision-making (Thorpe et al., 1983; Tremblay and Schultz, 1999; Elliott et al., 2000; Arana et al., 2003; Wallis, 2007, 2013; Bouret and Richmond, 2010; Noonan et al., 2010; Bachevalier et al., 2011; Rudebeck and Murray, 2011; Walton et al., 2011; Hampshire et al., 2012). This functional dichotomy between different OFC subfields has mostly been demonstrated using rewards and punishments, such as cues associated with food or monetary reinforcement. Yet, the OFC subfields may also play separable regulatory roles in the valuation of affective socioemotional cues, such as faces, that may serve as positive and negative reinforcers.

Humans and nonhuman primates are conspecific face experts and process faces using a combination of mutually non-exclusive scanning strategies, including holistic, configural, and feature-based processing (Parr, 2011, for review). Outside of the broader face processing network of the fusiform face area, superior temporal sulcus and amygdala (Rossion and Gauthier, 2002; Rolls et al., 2007; Dekowska et al., 2008; Tsao et al., 2008a), the OFC appears to play a regulatory role in face processing (Rolls, 2007; Tsao and Livingstone, 2008; Wright et al., 2008; Koski et al., 2017; Yankouskaya et al., 2017). Particularly, OFC areas 12 and 13 contain neurons that respond selectively to faces and voices (Thorpe et al., 1983; O’Scalaidhe et al., 1997; Tsao and Livingstone, 2008; Romanski and Diehl, 2011; Diehl and Romanski, 2014; Barat et al., 2018) and neuroimaging studies indicate that lOFC areas 12 and 13, but not vmOFC area 14, contain patches of face-selective neurons in the macaque prefrontal cortex (Tsao et al., 2008b). Further, some studies suggest that areas 12 and 13 may show differential activation to face stimuli (Tsao et al., 2008b) and face processing in these regions may be supported by separable processes (Zald et al., 2014). In humans, lOFC activity is modulated when attending to and judging emotion in faces (Vuilleumier et al., 2001; Monk et al., 2003) and proportionately increases with heightened intensity of facial expressions of anger (Blair et al., 1999). Similarly, damage to the lOFC is associated with reduced perception of emotions and the lOFC is specifically activated by negative emotions (Blair et al., 1999; Vuilleumier et al., 2001; Bramham et al., 2009; Golkar et al., 2012). Although these studies indicate that the lOFC may be more important than the vmOFC for attending to emotional faces, other studies in humans report impairments in processing emotional faces following broad lesions to ventromedial prefrontal cortex that included the OFC (areas 10, 11, 47) and medial PFC (areas 24, 25, 32), but not when the damage was limited to OFC areas 11, 13, and 47 (Jenkins et al., 2014). Furthermore, neuroimaging studies in humans have shown that the vmOFC is more active than the lOFC in the processing of faces relative to nonsocial positive stimuli (Troiani et al., 2016), building upon broad support for the vmOFC in the processing of appetitive cues (Tremblay and Schultz, 1999; O’Doherty et al., 2001; Bouret and Richmond, 2010). These contradictory findings on the role of the OFC subfields in processing socioemotional stimuli may have resulted from the different procedures used to assess attention to affective social stimuli and the type of emotional stimuli used (negative vs. positive), however few studies have directly compared separable lesions to OFC subfields under controlled conditions. Thus, there is a need to further explore the role of the OFC subfields in the modulation of attention to affective social stimuli.

To this end, we examined how rhesus monkeys (*Macaca mulatta*) with damage to specific OFC subfields visually explored scenes of conspecifics expressing different facial expressions (positive, neutral or negative) as compared to nonsocial stimuli of similar valence. Because the processing of faces and emotional expressions in primates is associated with autonomic physiological changes (Steinhauer and Hakerem, 1992; Lang et al., 1993; Bradley et al., 2001, 2008; Conway et al., 2007; Bramham et al., 2009; Laine et al., 2009; Skwerer et al., 2009), which have been associated with the vmOFC rather than lOFC (Nagai et al., 2004), we included an exploratory analysis of variations in pupil diameter while the monkeys were spontaneously scanning the images as a measure of autonomic arousal. Recently, we reported the effects of restricted but combined damage to OFC areas 11 and 13 on the processing of social and emotional images (Goursaud and Bachevalier, 2020). Compared to sham-operations, combined aspiration lesions of areas 11/13 increased the overall spontaneous scanning of faces, including a specific increase in exploration of the eye region. Although the ability to distinguish emotional from neutral faces was spared by OFC lesions, scanning patterns, especially of negative facial expressions (threats) were altered. Additionally, when viewing emotional conspecific faces, OFC-lesioned monkeys displayed increased pupil diameter that differed according to emotion type and face orientation. Yet, this study did not compare visual scanning patterns after damage to the most lateral (area 12) and the most medial (area 14) OFC subfields raising the possibility that the reported changes may not be restricted to damage to areas 11/13 but may also be found following damage of areas 12 and 14, as the aspiration lesions used in this study may have damaged connectional fibers between the most lateral and medial OFC subfields (Carmichael and Price, 1994). Thus, in the present study, we extended these earlier findings with an investigation of selective damage to three separate subfields of the OFC (areas 12, 13, and 14) on visual scanning patterns and processing of social cues (faces) and nonsocial cues (scenes) of different valence (negative, neutral and positive) embedded in dynamic, engaging movies, using more restricted lesions produced by neurotoxin injections that spared fibers “en-passage”. We first compared how monkeys with OFC lesions and controls modulated their visual scanning depending on the valence of social and nonsocial scenes. Based on the literature reviewed above, we predicted that emotional modulation would be altered by all OFC lesions for both social and nonsocial scenes. Given the presence of face-selective neurons within the lateral OFC network as compared to the ventromedial OFC network (Thorpe et al., 1983; O’Scalaidhe et al., 1997; Tsao et al., 2008b), we predicted that damage to areas within the lateral OFC network (areas 12 and 13) will yield more profound attentional impairments to social compared to nonsocial scenes than damage to the ventromedial OFC network (area 14). Further, given the presence of face-specific neurons in both areas 12 and 13 and that face processing in these regions may be supported by separable processes (see above), we predicted that lesions to each area will produce impairments to social attention independently, and perhaps differently. In addition, given some evidence that the lateral subfields are more active in the processing of negative stimuli than positive stimuli, and the broad support for the medial OFC in processing rewarding cues, we predicted that damage to lateral areas 12 and 13 will result in more profound impairments on attention to negative rather than positive stimuli, and vice-versa for damage to the medial area 14. Finally, given the strong connection of medial OFC area 14 to the regulation of autonomic physiological responses, we predicted that damage to the more medial OFC subfield, rather than the lateral OFC, will alter pupil size.

## Methods

All protocols and procedures were reviewed and approved by the Institutional Animal Care and Use Committee at Emory University and aligned with the standards set by the NIH Guide for the Care and Use of Laboratory Animals.

### Subjects

Seventeen rhesus macaques between the ages of 6 and 14 years of age (mean = 9.2) participated in this study. All subjects were born into large social groups at the Yerkes National Primate Center Field Station (Lawrenceville, GA, USA). Subjects were moved to the Yerkes National Primate Center Main Station (Atlanta, GA, USA) as adults and singly housed in appropriate cages allowing visual and auditory contacts with similarly aged macaques of both sexes. All subjects were fed a diet of nonhuman primate chow (Purina, St. Louis, MO, USA), given fruit and vegetables daily, and water *ad libitum*. All animals were trained for behavioral testing for related experiments (Kazama and Bachevalier, 2013), but were naïve to the procedures and stimuli presented in this experiment. Subjects were pseudorandomly assigned into one of four groups: lesions of OFC area 12 (Group OFC12, *n* = 4; one female), lesions of OFC area 13 (Group OFC13, *n* = 4, one female), lesions of OFC area 14 (Group OFC14, *n* = 5, four females), and sham-operations (Group C, *n* = 4, three females).

### Neuroimaging and Surgery

Neuroimaging procedures were performed the day of surgery and then again 1 week post-surgery for all animals in groups OFC12, OFC13, and OFC14. Vital signs and hydration were monitored and managed as subjects were sedated using ketamine HCl (10 mg/kg, 100 mg/ml), intubated, anesthetized with inhaled isoflurane (1.0 –2.0%, v/v, to effect), and placed into a stereotaxic apparatus using appropriate pain management treatment. Then, T1- [3D T1-weighted fast spoiled gradient (FSPRG)-echo sequence, TE = 2.6 ms, TR = 10.2 ms, 25° flip angle, contiguous 1 mm sections, 12 cm FOV, 256 × 256 matrix] and Fluid Attenuated Inversion Recovery [TE = 140 ms, TR = 1,000 ms, inversion time (TI) = 2,200 ms, contiguous 3 mm sections, 12 cm FOV, 256 × 256 matrix, acquired in three series offset 1 mm posterior] MR-images were acquired using a 3T Siemens Magnetom Trio system (Siemens Medical Solutions, Malvern, PA, USA) and a 5 inch coil to guide lesion placement and for later lesion estimation, respectively.

Following the pre-surgical neuroimaging session, all animals in the lesion groups were maintained under gas anesthesia, immediately transferred to the surgical suite, and prepared for surgical procedures using aseptic techniques. Following injection of Bupivicaine (0.25% concentration, 1.5 ml) along the location of the skin incision, the skin was cut from the mid-orbital ridge to the occiput and was thereafter retracted along with the temporal muscles underneath. A small craniotomy was made on the cranial bone at the level of the OFC in each hemisphere. The dura was retracted and a 30-gauge needle attached to a 10 μl Hamilton syringe was used to inject the neurotoxin. For injections of areas OFC13 and OFC14, the syringes were attached to stereotaxic arms and lowered based on stereotaxic coordinates estimated from the structural T1 images and bilateral surgeries were created simultaneously for all OFC13 and OFC14 lesions. For injections of OFC12, the syringes were handheld by the surgeon and bilateral lesions were created one at a time for each hemisphere in a one-stage surgery, except for one subject (OFC12-1), which received surgeries in the left and right hemispheres in a two-stage surgery 1 month apart. For all injections, ibotenic acid (0.8–1 μl; Biosearch Technologies, Novato, CA, USA, 10 mg/ml in PBS, pH 7.4) was delivered at a rate of 0.2 μl per min at each injection site. At the end of each injection, the needles were maintained in place for 1-min before their retraction to reduce spread of the neurotoxin across other brain tissue.

**Figure 1:**
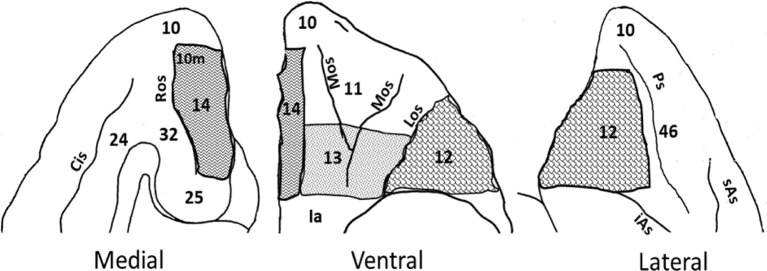
Intended orbitofrontal lesions. Illustration of the medial, ventral and lateral views of the orbitofrontal cortex (OFC). The intended orbitofrontal lesion areas include OFC12 (shingle), OFC13 (confetti), and OFC14 (zig zag). Surrounding areas of the OFC include on the ventral surface [OFC10 and insula (Ia)], on the medial surface (OFC10, 10m, 24, 25, and 32), and on the lateral surface (OFC10, 46). Abbreviations: Cis: Cingulate sulcus; iAs: Inferior arcuate sulcus; Ps: Principal sulcus; Los: Lateral orbital sulcus; Mos: Medial orbital sulcus; Ros: Rostral sulcus; sAs: Superior arcuate sulcus.

**Figure 2:**
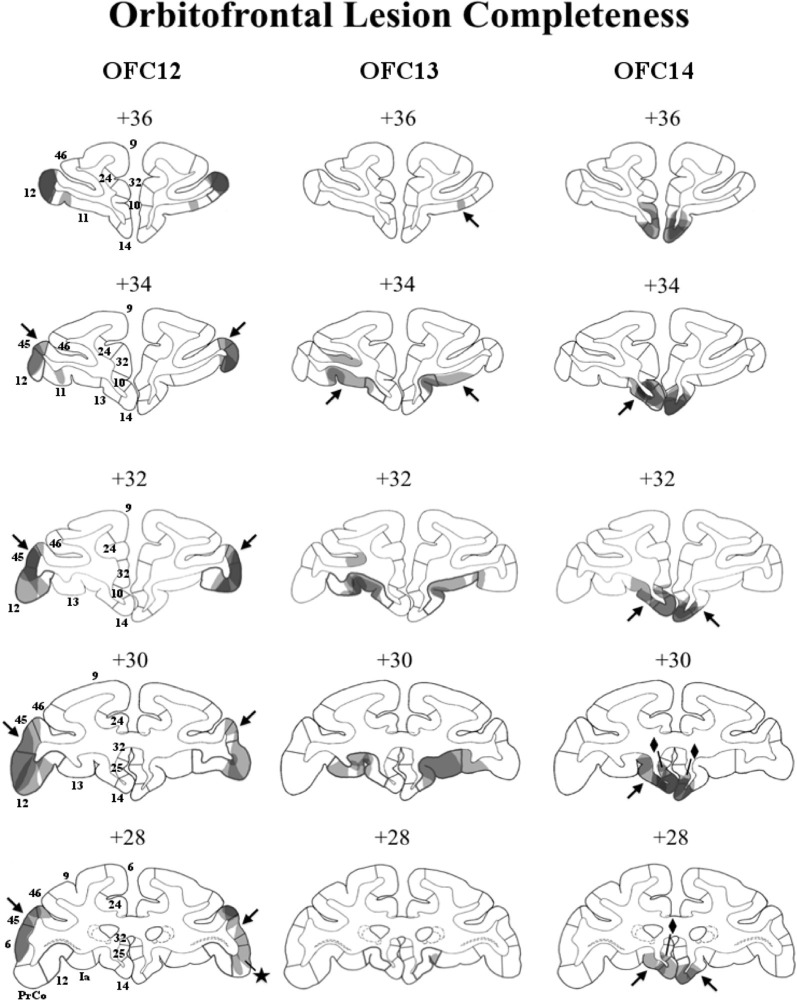
Lesion extent (cell loss) for each lesion group. Coronal sections through the extent of OFC12 lesions (left column), OFC13 lesions (middle column), and OFC14 lesions (right column). The bold outlines on each section indicate the extent of the intended lesions and numerals above each section refer to the distance in millimeters from the interaural plane. Lesion extent for each subject was layered on top of each other, such that darker areas indicate areas of damage common to all subjects, whereas lighter areas indicate areas of damage found in only some subjects. Unintended damage included area 45 (arrows) and PrCO (star) from Group OFC12; area 11 for Group OFC13 (arrows); areas 25 (diamonds) and 13 (arrows) for Group OFC14.

The extent of each intended lesion is illustrated on the ventral view of the OFC in Figure 1 and outlined with a bold line on the coronal sections of Figure 2. Bilateral lesions of OFC12 (4–23 injections) extended from few millimeters below the principal sulcus laterally to the fundus of the lateral orbital sulcus medially, and from the tip of the medial orbital sulcus anteriorly to posterior tip of the lateral orbital sulcus posteriorly. Bilateral lesions of OFC13 (seven injections) extended from the fundus of the lateral orbital sulcus laterally to the lateral border of the olfactory stria medially. The antero-posterior border of OFC13 lesions began from a virtual line drawn half-way from the anterior and posterior tips of the lateral and medial orbital sulci. Bilateral lesions to OFC14 (four to five injections) extended from the medial border of the olfactory stria medially to the rostral sulcus on the medial surface and from the anterior tip of the medial orbital sulcus anteriorly to the level of the posterior tip of lateral sulcus posteriorly.

For sham-operations, animals in Group C were sedated using ketamine HCl, intubated, anesthetized with inhaled isoflurane, and placed into a stereotaxic apparatus using appropriate pain management treatment in the surgical suite. As with lesioned subjects, sham-operated animals were maintained under gas anesthesia, and prepared using aseptic techniques. Then, after Bupivicaine was injected under the skin along the incision line, the skin was cut and the skin and muscle retracted, and a small craniotomy was made on the cranial bone at the level of the OFC in each hemisphere. In contrast to animals in Groups OFC12, OFC13, and OFC14, for sham-operations no needle was inserted and no injections were made.

For all subjects, once surgical procedures were complete, the wound was sutured in anatomical layers, and the subject was recovered from anesthesia. Post-surgical care included management of pain (acetaminophen, 10 mg/kg, p.o.), of infection (Cefazolin, 25 mg/kg i.m.), and of swelling due to immunoreactive process (dexamethasone sodium phosphate, 0.4 mg/kg i.m.). Recurrent monitoring of the animals was performed by veterinary and laboratory staff, for a minimum of 1 week post-surgery.

### Eye-Tracking Procedures

Eye position was measured using a 60 Hz infrared eye-tracker (ISCAN ETL400, Woburn, MA, USA) and a specially configured desktop computer. Images were presented on a 20^′′^ monitor using a custom stimulus presentation script in Presentation (v16.5; Neurobehavioral Systems, Inc., Berkeley, CA, USA). Subject gaze behavior was monitored on a second computer monitor by the experimenter during testing. Subjects were seated in a primate chair and wore a thermoplastic helmet to reduce head movements and maintain animals’ gaze toward the screen, thus reducing the testing time (Machado and Nelson, 2011).

#### Stimuli

All stimuli consisted of digital movies of monkeys and scenes. All movies were 720 × 480 pixel avi’s, unedited, and 10-s in length. For each subject, movies were shown across two testing sessions, roughly 6 months apart, and elicited similar levels of attention across both sessions (i.e., no significant differences in attention between session 1 and session 2, *z* = −1.64, *p* = 0.10). Each testing session contained three unique movies in each of category (positive social, neutral social, negative social, positive nonsocial, neutral nonsocial, negative nonsocial) for a total of 18 movies per session.

**Figure 3:**
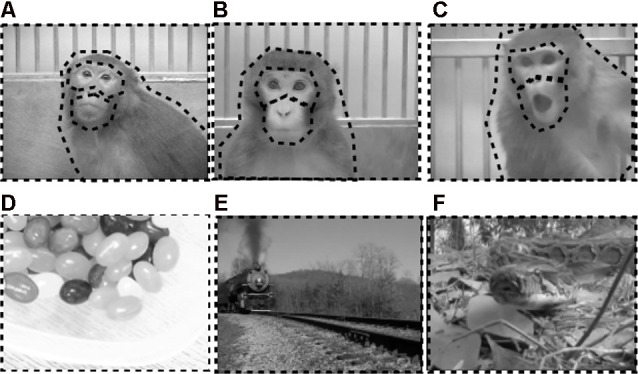
Examples of stimuli used in eye-tracking experiment. Top row depicts exemplars of still images taken from social movies of positive **(A)**, neutral **(B)**, and negative **(C)** valence. Bottom row depicts exemplars of still images taken from nonsocial movies of positive **(D)**, neutral **(E)**, and negative **(F)** valence. Dotted black borders outline the regions of interest (ROI) analyzed for each stimulus type. For social stimuli, ROIs covered the eyes, mouth, body, and whole scene. For nonsocial stimuli, ROIs covered the whole scene.

Social scenes depicted unknown adult rhesus macaques filmed through a clear panel in a holding cage. The six different movies in each emotional category depicted monkeys expressing either positive facial expressions (i.e., lipsmacks; Figure 3A), neutral expressions (Figure 3B), or negative facial expressions (i.e., open mouth threat; Figure 3C). Movies depicting emotional valence displayed periods of the specific facial expressions intermixed with periods of no facial expressions, and varied in monkeys’ movements within the cage and gaze direction.

Nonsocial scenes were pulled from online locations (e.g., YouTube) or recorded in the lab as necessary. All nonsocial scenes were judged to be absent of any face-like configurations and absent of any human or nonhuman primate feature (e.g., arm, foot) by an experimenter. In an effort to decouple the impacts of emotion and social content on attention to scenes, efforts were made to include nonsocial scenes that contained similar elements to the social scenes. This included the presence of movement, such that all six different movies in each nonsocial emotional category contained an object or non-primate animal moving in front of a discernible background. Positive nonsocial scenes included familiar food items or toys that had gained positive valence during previous cognitive testing (i.e., candy, fruit, chow, familiar toys; Figure 3D). Neutral nonsocial scenes included novel scenes of vehicles with no apparent or empirically validated valence or relevance to rhesus macaques (i.e., train, boats, bicycle, cars; Figure 3E). Negative nonsocial scenes included familiar and novel items known to elicit fear or threat response in monkeys. The familiar negative items were items used by veterinarians in the facility that have been judged to have negative associations (i.e., syringes). Novel negative items were movies of reptiles and invertebrates that have been shown to elicit fearful responses in both feral and lab reared macaques (i.e., snakes, tarantulas; Figure 3F; Nelson et al., 2003), including macaques with excitotoxic lesions to the OFC (Rudebeck et al., 2013).

#### Behavioral Testing Procedures

Subjects were transported from their home cage to a familiar behavioral testing room and transferred to a primate chair. They were then brought to the eye-tracking room and positioned between 18- and 24-inches in front of the testing monitor. Thermoplastic helmets were fitted and attached to the primate chair, and the subject was rewarded with preferred food items for cooperation. At the start of each testing session, eye location was calibrated by presenting a GIF animation in one of five preset positions on the screen (top left, top right, bottom left, bottom right, center). When the subject made a visible saccade to the location of the stimulus, the experimenter pressed a key to record the eye location in the ISCAN program. Successful calibration was assessed by presenting calibration stimuli during a “data-out” visualization mode in which a white crosshair on the screen represented the location of the subject’s gaze. If the crosshair matched the location of the calibration stimulus, calibration for that point was judged accurate. If the crosshair did not match, calibration procedures were repeated until all five calibration points appeared accurate. Eye-tracking calibration was completed within 10-min of arriving in the eye-tracking room. If an accurate calibration was obtained in less than 10-min, subjects remained calmly watching nature videos until 10-min had elapsed to ensure that all subjects were under the testing conditions for the same duration. All subjects were successfully calibrated during their first testing session, however in one case (ORB13-1 session 1) a power outage occurred mid-session and the testing was rescheduled and repeated on a later date. Ten-minutes following accurate calibration, the experimental stimuli were presented in a controlled, randomized order using Presentation software. All stimuli were presented for a total of five times, with a 30-s intertrial interval during which a small orienting GIF animation was presented at the center of a black screen. To ensure that calibrations did not change during testing, the experimenter monitored alignment between the gaze crosshair and orienting GIF animation between trials. If the calibration was no longer accurate, testing was cancelled, the subject was returned to its home cage, and the session was re-run on a later date. At the completion of stimuli presentation, the experimenter removed the thermoplastic helmet and rewarded the subject with both verbal and food reinforcement. Subjects were then returned to their home cage.

### Histological Lesion Assessment

Once all behavioral testing was complete, all subjects in the OFC groups were euthanized and perfused for post-mortem histological assessment of cell loss and quantification of lesion extent. Briefly, subjects were sedated and administered a lethal dose of sodium pentobarbital, and then perfused intracardially with saline and paraformaldehyde. Brains were then fixed in a 30% sucrose-formalin solution, followed by a cryoprotective solution, and then placed in a −80°C freezer until processed. Brains were cut frozen in the coronal plane at 50 μm. Every five brain sections (250 μm) throughout the extent of the OFC were stained with thionin (cell body) and every 20 brain sections (1 mm) were stained with gallyas (fiber). Each brain section was mounted on a glass slide, stained, and cover slipped. Slides were then magnified at 0.28× and digitized using a Leica Z6 Microscope (Leica Microsystems, Wetzlar, Germany) fixed with a Excelis HD Lite Camera (Accu-Scope Inc., Commack, NY, USA). The digitized coronal sections were matched with those of an atlas of a normal monkey brain and areas of cell loss within the anterior-posterior extent of each area and cell loss within cortical layers were drawn onto control sections using Photoshop (v8; Adobe Systems Inc., San Jose, CA, USA). The resulting image of cortical cell loss in atlas space was then quantified using Image-J software (v1.46r; U.S. National Institutes of Health, Bethesda, MD, USA), and cell loss in both intended and unintended regions was recorded and calculated as a percent of the normal control brain. Thus, cell loss in specific OFC subfields could either occupy the entire subfield and all cortical areas, or could vary according to its anterior-posterior extent and within cortical layers.

### Data Analysis

Raw eye-tracking data and raw stimulus presentation data were combined in Excel (Microsoft, Redmond, WA, USA). All data were then imported into the GazeTracker (v10.0; Eyetellect, Charlottesville, VA, USA) program for analyses. Using GazeTracker, frame-by-frame regions-of-interest (ROIs) were drawn on predetermined salient regions of the stimuli and adjusted in size and location using a combination of manual editing and digital interpolation to account for the movement of regions throughout the duration of the video. For social stimuli, ROIs were drawn on the eyes, mouth, body (inclusive of eyes and mouth) and whole scene (see Figures 3A–C for ROIs on social videos). For nonsocial stimuli, a single ROI was drawn around the whole scene. Gaze points beyond the whole scene were not analyzed. Both frequency and duration of fixation were analyzed and provided similar results, so only fixation duration will be reported below.

To ensure validity of comparisons across ROIs the following comparisons were performed. To control for overall looking time, attention to ROIs was expressed as a proportion of total time attending to the ROI divided by total time attending to the whole scene of the same movie. Average ROI size of the eyes did not significantly differ from the ROI size of the mouth (*Z* = −0.16, *p* = 0.74). To determine whether attention to the mouth was restricted to times when emotional expressions were present in the videos, we conducted a *post hoc* comparison of attention to the eyes and mouth when an expression was present separately from times when no expression was present. Regardless of whether an emotional expression was present or not, attention to the eyes was higher than attention to the mouth (Exp. Eyes > Exp. Mouth: *Z* = −2.43, *p* = 0.02; No Exp. Eyes > No Exp. Mouth: *Z* = −3.62, *p* < 0.001). In addition, subjects spent significantly less time attending to the eyes when an emotional expression was present (Exp. < No Exp.: *Z* = −2.77, *p* < 0.01) and subjects tended to spend less time attending to the mouth when an emotional expression was present (Exp. < No Exp.: *Z* = −1.77, *p* = 0.08). The greater attention to eyes and mouth during periods containing no facial expression may be due to the ambiguity presented by absence of any clear emotional expression.

In addition to eye tracking analysis, exploratory analysis of average pupil diameter (in pixels) was included to compare the effects of OFC lesions on arousal to emotional stimuli, as reported earlier (Goursaud and Bachevalier, 2020). Average pupil diameter (in pixels) was captured using the infrared eye-tracking camera while subjects viewed the 10-s long social and nonsocial emotional videos. No baseline data was recorded outside of stimulus presentation, so to exclude the potentially confounding effects of the pupillary light response at stimulus onset, the first 500 ms of stimulus presentation were not included in the pupil analysis.

All data were first tested for normality and homogeneity of variance. We used a mix of parametric and non-parametric analyses as appropriate.

First, to determine the role of OFC subregions in the processing of social compared to nonsocial scenes, we used the Wilcoxon signed-rank test to compare the main effect of social context on total looking to each video. We then used the Mann–Whitney *U* test to directly compare each treatment group across social context.

Next, to determine the role of OFC subregions on the processing of emotional cues, we used the Friedman test to compare the main effect of valence on total looking to each video regardless of social context. Where appropriate, Wilcoxon signed-rank tests were used to compare the effects of each valence condition. We then used the Mann–Whitney *U* test to directly compare each treatment group across valence. In the case where social and nonsocial attention differed, we then analyzed how damage to OFC subregions impacted attention to emotional cues separately for social and nonsocial contexts. To do so, we used the Friedman test to separately compare the main effects of valence within each social context and then used Wilcoxon signed-rank tests to compare effects of each valence condition and the Mann–Whitney *U* test to directly compare each treatment group.

To determine the effects of OFC damage on attention to social emotional cues, we compared the percent of time spent attending to the body of the monkey relative to the total time spent attending to the video. We used the Friedman test to compare the main effect of valence on percent attention to the body of social stimuli. Where appropriate, Wilcoxon signed-rank tests were used to compare the effects of valence and the Mann–Whitney *U* tests were used to directly compare each treatment group across valence.

Given that in our previous study (Goursaud and Bachevalier, 2020) combined OFC11/13 lesions produced specific alterations to attention to the eyes of social stimuli, we compared percent of attention to the eyes of social stimuli across valence. To do so, we used the Friedman test to compare the main effect of valence. Where appropriate, Wilcoxon signed-rank tests were used to compare the effects of each valence condition and the Mann–Whitney *U* tests were used to directly compare each treatment group across each valence.

To determine whether changes in attention to the eye region could be driven by general attention to the face region, we also compared percent of attention to the adjacent mouth region of social stimuli across valence. To compare attention to the mouth, we used a two-way (Group × Valence) RM-ANOVA with Group as the between-subject factor and Valence as the within-subject repeated factor.

Last, to understand the involvement of OFC subregions on the regulation of autonomic arousal toward social and nonsocial contexts across emotional valence, we used exploratory analysis of the average pupil diameter across stimulus presentation. A three-way (Group × Social Context × Valence) RM-ANOVA was used to compare pupil diameter with Group as the between-subject factor and Social Context and Valence as the within-subject repeated factors. When pupil diameter differed between social and nonsocial scenes, a two-way (Group × Valence) RM-ANOVA was used to compare pupil diameter separately for social and nonsocial stimuli.

To determine whether the sex of the subject impacted attention to social and nonsocial stimuli *post hoc*, exploratory *t*-tests were used to compare average attention or percent of attention between males and females across all variables. *Post hoc* Bonferroni corrected tests were used to compare the effects of either Group, Social Context or Valence when significant. Additionally, because of the small sample size and unequal males and females within each group, when interactions between factors were not significant, planned comparisons were performed between the control group and the experimental groups, using one-sided planned comparisons (Pedhazur, 1982), since this comparison provides more statistical power against type II error, i.e., not rejecting the null hypothesis when it is false. For all analyses, a *p* < 0.05 was considered significant, and effect sizes (partial eta squared) were calculated for parametric tests. Finally, due to the small sample size for each group, we did not run correlation between behavioral data and lesion extent. However, for Groups OFC13 and OFC14, we ran the statistical analyses with and without the animals with the smallest percent of cell loss (e.g., OFC13-1 and OFC13-3 and OFC14-3) and did not find a different pattern of results, thus these animals were included for all statistical analyses reported below.

## Results

### Description of Lesion Assessment

A visualization of lesion extent (cell loss) for each area is illustrated in Figure 2 and percent of cell loss is given for each animal of the three OFC groups in Supplemental Table S1. On Figure 2, for each OFC group, lesion extent for each subject was layered on each other, such that darker areas indicate area of damage common to all subjects, whereas lighter areas indicate damage to fewer subjects. It is important to note that for some cortical areas the cell loss did not include all layers of the cortex, resulting in overall small lesion extent. Yet, damage to only some cortical layers likely resulted in a dysfunction of the entire cortical region. Thus, although the percent of cell loss in each OFC region appears small, it resulted in functional disruption far greater than what the percent cell loss indicates.

In Group OFC12, average bilateral cell loss varied between 15% and 41%, with three animals (OFC12-1, OFC12-2, OFC12-3) showing bilateral symmetrical lesions (Left hemisphere average: 27% and Right hemisphere average: 23%). Animal OFC12-4 had a more unilateral lesion (Left: 26%, Right: 4%), with the lesion missing the posterior two-thirds of OFC area 12 on the right side. As shown in Figure 2 (left column), for all three cases with symmetrical lesions as well as the left hemisphere of case OFC12-4, the extent of cell loss was found in all cortical layers and covered the entire anteroposterior extent of area 12, especially on the left hemisphere, but spared the most ventrolateral portion of area 12. All OFC12 cases received minor unintended damage to area 45 (range: 10–31%; see Figure 2, left column, arrows in +34, +32, +30, and +28), and cases OFC12-1, OFC12-3, and OFC12-4 received minor damage to PrCO (range: 1–9%; see Figure 2, left column, star at +28).

In Group OFC13, average cell loss varied from 2% to 26% with two animals (OFC13-2, OFC13-5) receiving bilateral symmetrical lesions, two animals (OFC13-3 and OFC13-4) receiving more unilateral lesions (OFC13-3-L: 14%, R: 0%; OFC13-4-L: 12%, R: 27%), and one animal OFC13-1 receiving only a small (2%) lesion. As shown in Figure 2 (middle column), the cell loss was visible across the entire anteroposterior extent of area 13 with some sparing of the most posterior portion of area 13 for all cases except OFC13-1. In addition, the cell loss was observed mostly in the superficial cortical layers (Layers I–III) for four cases (OFC13-2 to OFC13-5), but varied in location from case to case through the entire extent of area 13. Cases OFC13-2, OFC13-3, and OFC13-5 received unintended damage to area 11 (1–20%; see Figure 2, middle column, arrows at +36 and +34 bilaterally).

In Group OFC14 (Figure 2, right column), average cell loss varied between 1 and 41%, with two animals (OFC14-1 and OFC14-2) receiving bilateral symmetrical lesions (L: 47%, R: 34.8%), one animal (OFC14-4) receiving a more unilateral lesion (L: 5%, R: 47%) and one animal OFC14-3 receiving a small (<1%) lesion. This cell loss spanned the full anteroposterior extent of area 14 for all animals except OFC14-3, but spared the ventral tip of area 14 for some animals. All animals received unintended damage to area 13 (4–20%; see Figure 2, right column, arrows at +34, +32, +30, and +28) and area 25 (7–16%; see Figure 2, right column, diamonds at +30 and +28) mostly restricted to the left hemisphere.

### Effects of OFC Lesions on Attention to Social and Nonsocial Stimuli

**Figure 4:**
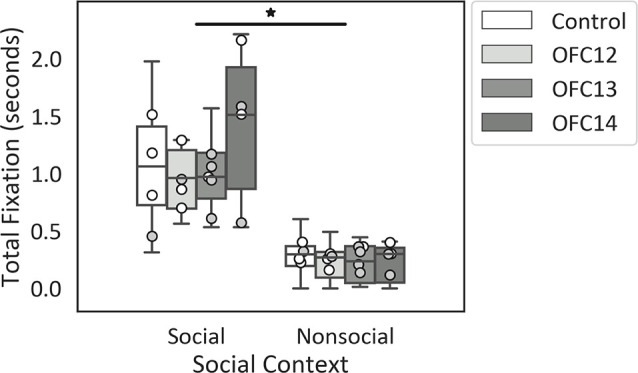
Effects of OFC lesions on attention to social and nonsocial stimuli. Scores are total fixation in seconds to social and nonsocial scenes for the control animals (Control), animals with damage to OFC area 12 (OFC12), animals with damage to OFC area 13 (OFC13), and animals with damage to OFC area 14 (OFC14). Attention to social scenes was greater than attention to nonsocial scenes. None of the planned comparisons between treatment groups reached significance for total looking to social or nonsocial stimuli (all *p*’s > 0.05). Scores of males are shown in open circles and those of females in gray circles. *p < 0.05.

Focal lesions of OFC12, OFC13, or OFC14 did not impact the modulation of attention to social over nonsocial stimuli. Regardless of treatment group, all animals spent more time looking at the entire social scenes as compared to nonsocial scenes (*Z* = −3.62, *p* < 0.001; Figure 4). None of the planned comparisons between treatment groups reached significance (all *p*’s > 0.05).

### Effects of OFC Lesions on Attention to Emotional Stimuli

**Figure 5:**
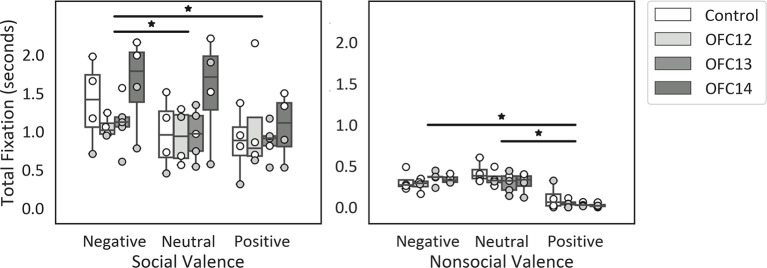
Effects of OFC lesions on attention to valenced social and nonsocial stimuli. Scores are total fixation in seconds for social scenes (left) and nonsocial scenes (right) for each valence (Negative, Neutral, and Positive). For social scenes, attention to negative scenes was greater than attention to neutral and positive social scenes (left) and for nonsocial scenes, attention to negative and neutral nonsocial scenes was greater than positive nonsocial scenes (right). None of the planned comparisons between treatment groups reached significance for total looking to positive, neutral, or negative scenes (all *p*’s > 0.05). *p < 0.05. Conventions as in Figure 4.

Given that overall attention was significantly different for social and nonsocial contexts, we compared the effect of OFC lesions on attention to emotional cues within each social context separately (see Supplemental Table S2A for individual scores). Within social stimuli, subjects fixated longer on the negative scenes compared to neutral and positive scenes ($\chi_{\left(2\right)}^{2}$ = 9.88, *p* < 0.01; Neg > Neu: *Z* = −2.06, *p* = 0.04; Neg > Pos: *Z* = −2.82, *p* < 0.01; Figure 5, left). Within nonsocial stimuli, subjects fixated longer on the negative and neutral scenes compared to positive scenes ($\chi_{\left(2\right)}^{2}$ = 22.71, *p* < 0.001; Neg > Pos: *Z* = −3.57, *p* < 0.001; Neu > Pos: *Z* = −3.62, *p* < 0.001; Figure 5, right). None of the planned comparisons between treatment groups reached significance (all *p*’s > 0.05).

Although the number of males and females in each group were very small, we performed *post hoc* exploratory *t*-tests to determine whether the sex of subjects impacted attention to social and nonsocial scenes and found that when all groups were considered together there were significant effects of sex on total fixation to positive and negative social scenes and to neutral nonsocial scenes. Specifically, male monkeys looked longer at positive and negative social scenes than female monkeys (Pos. Social Scene: *t*_(15)_ = 2.23, *p* = 0.04; Neg. Social Scene: *t*_(15)_ = 2.84, *p* = 0.01), and also looked longer at neutral nonsocial scenes (Neu. Nonsocial Scene: *t*_(15)_ = 2.33, *p* = 0.03). No other comparisons reached significance. This appears to be most pronounced for Groups Controls and OFC14 (see Figure 5, Supplemental Table S2A), and given the small sample size and unequal sex ratio across groups, it was not possible to determine if these differences are driven by outliers or fundamental differences between sexes in visual attention.

### Effects of OFC Lesions on Attention to the Body of Social Stimuli

**Figure 6:**
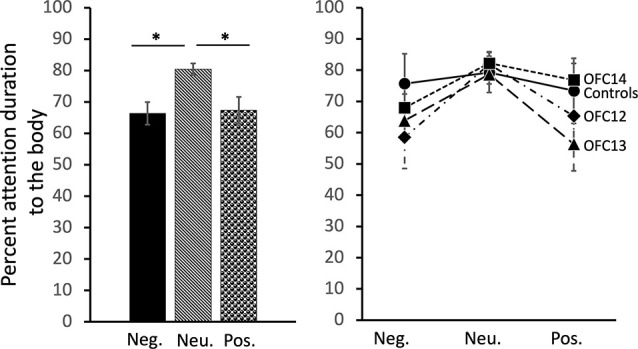
Effects of OFC lesions on attention to the body of social stimuli. Scores are percent fixation duration to the body of social stimuli. Regardless of treatment group, all animals tended to look at the body of neutral social stimuli for a greater proportion of time than they looked at the body of positive or negative social stimuli (left and right). *p < 0.05. Conventions as in Figure 4.

Focal lesions of OFC12, OFC13, or OFC14 did not impact the modulation of visual scanning of the body of the movie monkey across valence. Although subjects in all four groups spent an equal amount of time viewing the body of positive and negative social stimuli (*p* > 0.10), they spent a greater proportion of their time viewing the monkey body for neutral social stimuli (Main effect of valence: $\chi_{\left(2\right)}^{2}$ = 9.29, *p* = 0.01; Neu > Pos: *Z* = −3.01, *p* < 0.01; Neu > Neg: *Z* = −3.05, *p* < 0.01; Figure 6, left and Supplemental Table S2B for individual scores). This difference was more pronounced for animals in Groups OFC12 and OFC13 than for animals in Groups C and OFC14 (Figure 6, right), although none of the planned comparisons between treatment groups reached significance (all *p*’s > 0.05).

### Effects of OFC Lesions on Attention to the Eyes of Social Stimuli

**Figure 7:**
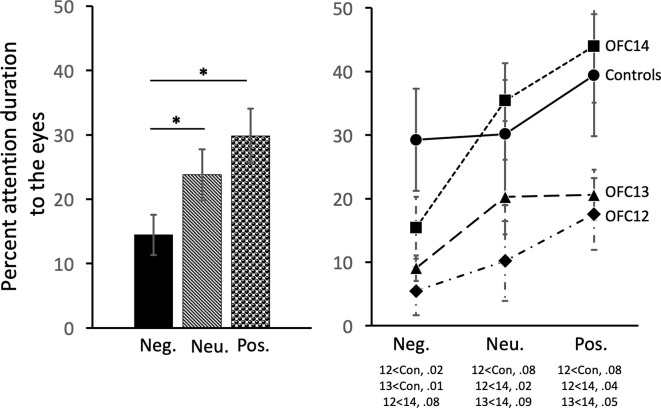
Effects of OFC lesions on attention to the eyes of social stimuli. Scores are percent fixation duration to the eyes of social stimuli. All animals showed a preference for viewing the eyes of positive and neutral social stimuli compared to negative (left). However, animals in groups OFC12 and OFC13 showed blunted attention to the eyes, particularly to the eyes of negative stimuli (right). *p < 0.05. Conventions as in Figure 4.

Subjects tended to pay less attention to the eyes of negative stimuli than to the eyes of neutral and positive stimuli ($\chi_{\left(2\right)}^{2}$ = 19.43, *p* < 0.001; Neg < Neu: *Z* = −2.38, *p* < 0.02; Neg < Pos: *Z* = −3.62, *p* < 0.001; Figure 7, left and Supplemental Table S2B for individual scores). Interestingly, though subjects in both Groups OFC12 and OFC13 did not differ from each other for any emotional valence (all *p*’s > 0.05), both attended less to the eyes of negative social stimuli (Figure 7, right) as compared to both control subjects and those with OFC14 lesions. This blunted attention to the eyes appeared more pronounced for Group OFC12 than Group OFC13, but this group difference did not reach significance (*p* > 0.05). Thus, as compared to controls, both Groups OFC12 and OFC13 displayed significantly less attention to the eyes of negative stimuli (OFC12 < Cont: *U* = 0, *p* = 0.02; OFC13 < Cont: *U* = 0, *p* = 0.01), yet only Group OFC12 showed a trend towards a significant decrease in attention to the eyes for neutral and positive stimuli (*U* = 2, *p* = 0.08 for all comparisons). The blunted attention to the eyes for Group OFC12 also reached significance for the positive and neutral stimuli as compared to Group OFC14 (*U* = 1, *p* = 0.04 and *U* = 0, *p* < 0.02, respectively), but only reached trend level for the negative stimuli (*U* = 2, *p* = 0.08). The comparison between Groups OFC13 and OFC14 showed a trend towards significance for the positive and neutral stimuli (*U* = 2, *p* = 0.05 and *U* = 3, *p* = 0.09, respectively), but not for the negative stimuli.

### Effects of OFC Lesions on Attention to the Mouth of Social Stimuli

**Figure 8:**
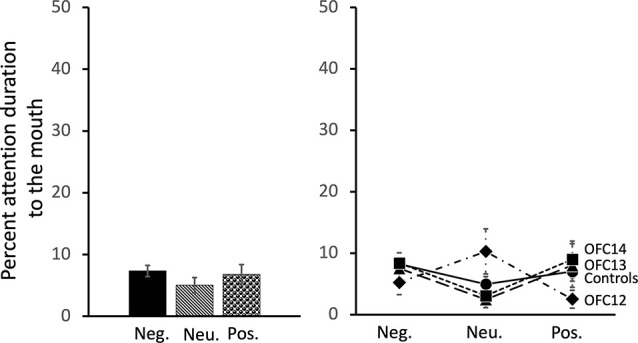
Effects of OFC lesions on attention to the mouth of social stimuli. Scores are percent fixation duration to the mouth of social stimuli. OFC lesions spared the distribution of attention to the mouth of social stimuli (right and left). Conventions as in Figure 4.

All subjects spent almost no time fixating on the mouth of social stimuli. Thus, in contrast to the effects of OFC lesions on attention to the eyes, there were no significant impacts of OFC lesions on percent attention to the mouth of social stimuli nor were there any effects of stimulus valence on percent attention to the mouth (Group: *F*_(3,13)_ = 0.13, *p* = 0.94, η^2^ = 0.03; Valence: *F*_(2,26)_ = 0.77, *p* = 0.48, *η*^2^ = 0.06; Valence × Group: *F*_(6,26)_ = 1.74, *p* = 0.15, *η*^2^ = 0.29; Figure 8 and Supplemental Table S2B for individual scores).

### Effects of OFC Lesions on Modulation of Autonomic Arousal to Social and Nonsocial Stimuli

Regardless of treatment group, all animals showed reduced pupil diameter when viewing social scenes compared to nonsocial scenes (*F*_(2,26)_ = 53.75, *p* < 0.001, *η*^2^ = 0.81). None of the planned comparisons between treatment groups reached significance (all *p*’s > 0.05).

### Effects of OFC Lesions on Modulation of Autonomic Arousal to Emotional Stimuli

**Figure 9:**
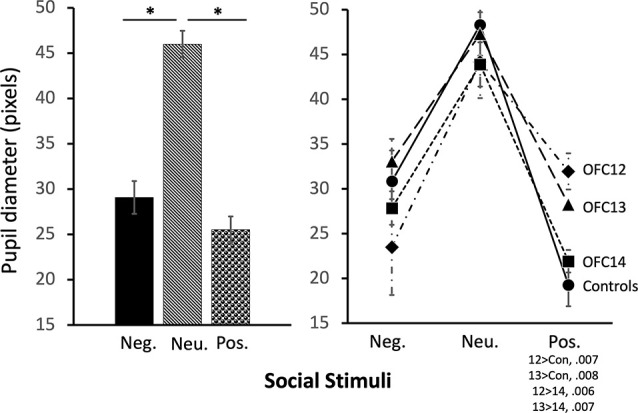
Effects of OFC lesions on modulation of autonomic arousal (pupil diameter) to social stimuli. Scores are pupil diameter in pixels. All animals showed decreased pupil diameter to negative and positive social scenes as compared to neutral social scenes (left). However, animals in groups OFC12 and OFC13 showed heightened arousal (greater pupil diameter) when viewing positive social scenes compared to controls and group OFC14 (right). *p < 0.05.

**Figure 10:**
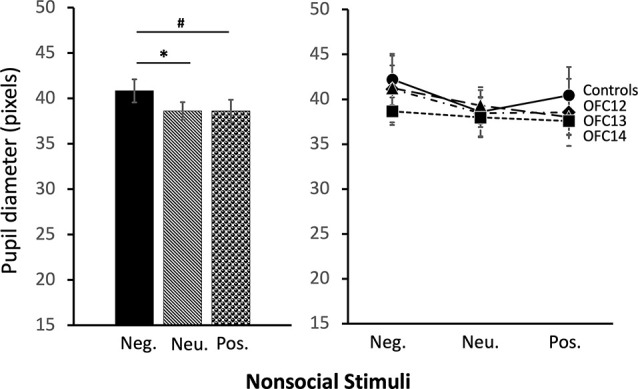
Effects of OFC lesions on modulation of autonomic arousal (pupil diameter) to nonsocial stimuli. Regardless of lesion group, animals showed increased pupil diameter when viewing negative nonsocial stimuli compared to neutral and positive nonsocial stimuli (left). *p < 0.05; ^#^*p* < 0.10.

The modulation of arousal to emotional valence differed for social and nonsocial scenes. For social scenes there was a main effect of valence, such that all subjects showed reduced pupil diameter in response to emotional compared to neutral scenes (*F*_(2,26)_ = 68.60, *p* < 0.001, *η*^2^ = 0.84; Pos < Neu: *p* < 0.001; Neg < Neu: *p* < 0.001; Figure 9, left and Supplemental Table S2C for individual scores). This effect significantly interacted with groups, such that for positive social scenes, subjects with OFC12 and OFC13 lesions had greater pupil diameter compared to both controls (*F*_(6,26)_ = 2.88, *p* = 0.03, *η*^2^ = 0.40; OFC12 > Control: *t*_(6)_ = 4.04, *p* < 0.01; OFC13 > Control: *t*_(7)_ = 3.64, *p* < 0.01; Figure 9, right) and OFC14 lesions (OFC12 > OFC14: *t*_(6)_ = 4.23, *p* < 0.01; OFC13 > OFC14: *t*_(7)_ = 3.76, *p* < 0.01). There were no significant differences in pupil diameter between groups for the negative and neutral social scenes or between Group C and Group OFC14 (all *p*’s > 0.05). For nonsocial stimuli, there was a main effect of valence such that subjects showed increased pupil diameter in response to negative compared to neutral scenes and a trend toward increased pupil diameter in response to negative compared to positive scenes (*F*_(2,26)_ = 4.60, *p* = 0.02, *η*^2^ = 0.26; Neg > Neu: *p* = 0.01; Neg > Pos: *p* = 0.09; Figure 10). However, there were no effects of treatment group on autonomic arousal to nonsocial scenes and there were no significant differences in pupil diameter between groups for any nonsocial scenes (all *p*’s > 0.05).

## Discussion

This study indicated that subregions of the OFC make separable contributions to social attention and social emotion regulation. Overall, all subjects attended more to social than nonsocial scenes and all subjects attended more to negative than to positive scenes, indicating that damage to OFC12, 13, and 14 spared the ability to modulate attention across social context (social, nonsocial) and emotional valence (negative, neutral, and positive). By contrast, as predicted from earlier literature, damage to the lateral OFC subfields (OFC12 or OFC13) fundamentally disrupted the distribution of attention to a critical region for social information, the eyes, whereas damage to the most medial OFC subfields (OFC14) did not. This finding appears to be unique to the socially relevant area of eyes, as damage to OFC12, 13, and 14 did not yield measurable alterations in attention to the mouth, a face area that also carries critical socioemotional information. Yet, contrary to our prediction, damage to the lateral OFC subfields (OFC12 and OFC13) did alter the regulation of autonomic arousal in the presence of positive emotional scenes, but damage to OFC14 alone did not. These findings will be discussed in turn for each OFC subfield.

### OFC Area 12 Lesions Impair Attention to Salient Social Cues and Arousal to Positive Social Cues

Lesions to OFC12 did not alter the preference to explore social scenes compared to nonsocial scenes or the modulation of visual scanning across emotional valence of social and nonsocial scenes. However, across all emotional valences of social scenes, three of the four OFC12 animals showed a blunted attention to the eyes as compared to controls, an effect that was particularly pronounced when viewing negative stimuli (Supplemental Table S2B, Figure 7). Furthermore, compared to control animals, all four animals with OFC12 lesions showed greater pupil diameter when viewing positive social scenes (Supplemental Table S2C, Figure 9), indicating altered modulation of arousal for positive social signals. Taken together, these findings suggest that dysfunction of OFC12 may yield subtle alterations in the processing of emotionally charged cues as reflected by an avoidance of salient regions, particularly the eyes of negative faces, and increased arousal to positive social stimuli.

Despite the small number of animals and the variability in lesion extent, the findings support our prediction that damage to OFC12 yields impairments in the processing of socioemotional cues. This finding is consistent with previous studies in humans and nonhuman primates suggesting that the lOFC is important for processing social affective cues by directing attention to salient social cues and mounting an appropriate emotional response. For example, in humans, OFC area 12 and the lateral OFC show specific functional activation to negatively valenced facial expressions compared to neutral ones during emotional face viewing tasks (Blair et al., 1999; Vuilleumier et al., 2001). In addition, in humans, extensive prefrontal lesions that included area 12 impaired the recognition of negative emotion in faces (Dal Monte et al., 2013), and yielded decreased eye gaze, especially to fearful faces (Wolf et al., 2014). Likewise, in macaques, the lateral OFC exhibits specific functional activity when monkeys view social interactions (Sliwa and Freiwald, 2017) and greater activation of OFC area 12 was reported when macaques viewed expressive faces vs. neutral faces (Tsao and Livingstone, 2008). A number of studies in marmosets indicate that damage to the lateral OFC is associated with increased anxiety in social (Agustín-Pavón et al., 2012) and nonsocial contexts (Shiba et al., 2015) due to impaired coping mechanisms (Shiba et al., 2015). Within the social contexts examined here, the finding of decreased eye attention following OFC12 lesions (see Figure 7) may reflect an increase in anxious behavior that resulted in an inability to direct attention and assign value to relevant, predictive cues (Shiba et al., 2016; Roberts, 2020). Although our study did not replicate the findings that lesions including area 12 impair nonsocial emotional processing as well (Shiba et al., 2015), this suggests that the general processing of nonsocial emotional cues in rhesus macaques is not limited to the lOFC area 12 and may be bolstered by emotion processing capabilities of nonsocial cues in other OFC regions, such as OFC 13 and OFC 14 (Pujara et al., 2019).

OFC12 lesions also increased subjects’ pupil diameter when viewing faces expressing positive valence. These results suggest that OFC area 12 plays a role in the self-regulation of emotional arousal in non-human primates. Although the sympathetic tone during emotion processing appears to be modulated by the amygdala (Adolphs et al., 1994, 2002; Laine et al., 2009; Terburg et al., 2012; de Gelder et al., 2014; Dal Monte et al., 2015), prefrontal cortex areas, including the OFC, exert a top-down regulation of the amygdala to enable cognitive reappraisal and effective emotion regulation (Bachevalier and Loveland, 2006; Johnstone et al., 2007; Roberts, 2020). For instance, in humans, down-regulation of negative emotion *via* reappraisal specifically activated the lateral orbitofrontal cortex and resulted in concurrent decrease in amygdala activity (Ochsner et al., 2004). In addition, decreased amygdala activity has been associated with effort-related pupil dilation (Urry et al., 2006; Johnstone et al., 2007; van Reekum et al., 2007). Thus, in the present study, the slight heightened pupil dilation in monkeys with OFC12 lesions may similarly reflect higher arousal resulting from an increased amygdala activity. In the present study, the impact of OFC12 lesions on alternations of autonomic arousal was limited to positive social cues, and not present for negative social stimuli. Though attention and arousal are often linked, the direction of their influence on animal’s response is not entirely clear. In our study, lesions of OFC12 resulted in decreased attention to the eyes of negative social stimuli but spared alterations in autonomic arousal when viewing the same negative cues. Thus, it is possible that the limited attention to the eyes of the negative social stimuli in animals with OFC12 lesions as compared to controls could have been insufficient to produce autonomic arousal (see Figure 7, right). In addition, lesions of OFC12 resulted in increased arousal when viewing positive social scenes, but only trend-level alterations in attention to the eyes of positive social stimuli. This difference between the impacts of OFC12 lesions on attention and arousal for positive social scenes may be related to a failure to assign appropriate value to emotional cues or differences in emotion regulation strategies. For example, our data showed that the heightened arousal observed when animals with OFC12 lesions view positive social scenes approached the same level of arousal observed when controls viewed negative social scenes (see Figure 7, right). Thus, the difference may be due to altered valuation of socioemotional cues resulting in the treatment of positive cues as being more negative. In humans, attention and arousal while viewing emotional social cues appear to be disrupted in individuals with heightened anxiety toward social cues, though the direction of the disruption is disputed. Some studies report increased attention to and inability to disengage from emotional social cues in individuals with social anxiety disorder (Wieser et al., 2009b; Buckner et al., 2010), whereas others report decreased attention (Mansell et al., 1999; Heuer et al., 2007; Schneier et al., 2011). One explanation may be due to the time-course of evaluation, where attention to threats is initially high in individuals with heightened anxiety, and then drops off as avoidance strategies are adopted. Though findings on the time-course of attention are mixed (Wieser et al., 2009a; Buckner et al., 2010; Schofield et al., 2012), these studies generally limit exposure to 1–3 s, compared to the total 10-s of stimulus presentation in our study. Future studies should investigate the temporal pattern of arousal changes linked to gaze direction while viewing ecologically relevant dynamic social stimuli to further examine this possibility. Overall, the findings support the important role of the lateral OFC area 12 in attention to social cues, particularly cues with a negative valence, and in the modulation of arousal.

### OFC Area 13 Lesions Impair Attention to Salient Social Cues and Arousal to Positive Social Cues

Similar to OFC12 lesions, selective lesions of OFC13 resulted in decreased attention to the eyes of social stimuli, particularly to the eyes of negative social stimuli, and heightened arousal when viewing positive social stimuli. Despite relatively small lesions that encroached mostly on the superficial cortical layers, all five animals with OFC13 lesions showed less attention to the eyes of negative stimuli and three of the five animals with OFC13 lesions also showed less attention to the eyes of positive stimuli as compared to controls (Supplemental Table S2B, Figure 7). Furthermore, all animals with OFC13 lesions displayed greater pupil dilation to positive social scenes (Supplemental Table S2C, Figure 9). As with lesions to OFC12, despite disrupted attention to the eyes and dysregulated autonomic arousal, the preference for viewing social scenes rather than nonsocial scenes was spared.

Despite individual variation in attention to stimuli and variations in lesion extent, these results were broadly consistent with current understanding of the lateral OFC area 13 in the modulation of emotion regulation, although notable inconsistencies between studies have been reported (see discussion below). For example, damage to the OFC in humans including area 13 impairs discrimination of emotional expressions from neutral faces (Tsuchida and Fellows, 2012), presumably due to impaired attention to the eyes. Monkeys with aspiration lesions of OFC13 and OFC11 display blunted response to social threats (Izquierdo et al., 2005; Kalin et al., 2007; Bachevalier et al., 2011; Kazama et al., 2014) and receive increased aggression and threatening behaviors from their partners, likely due to impaired emotional behavior exhibited by the OFC-lesioned animals towards their control peers. However, the absence of changes in attention to nonsocial threatening stimuli after OFC13 excitotoxic lesions contrasts with the enhanced anxious temperament of macaques in presence of nonsocial threat reported by Pujara et al. (2019). This difference may be due to the fact that in the Pujara study: (a) the excitotoxic OFC lesions were not restricted to OFC13 but included OFC11; (b) the stimuli (rubber snake) may have been more effective at eliciting a response to nonsocial threat stimuli than the snake videos shown in the present study; and (c) the methodology employed by Pujara did not exclude the possibility that anxious temperament was accompanied by avoidance of salient regions of the nonsocial negative stimulus. Further, the reduction of attention to the eyes after OFC area 13 lesions contrasted with those of a recent report (Goursaud and Bachevalier, 2020) that indicated an increased attention to the eyes while monkeys looked at static images of faces and face-like objects. This difference again may have resulted from: (a) the use of video clips instead of static images; and (b) the inclusion of OFC area 11 in addition to OFC area 13 and inadvertent damage to area 14 and fibers-in-passage resulting from aspiration lesions in the earlier study. Area 13 and area 11 share extensive interconnectivity (Carmichael and Price, 1996; Price, 2006) and likely work in concert to support stimulus valuation (Elliott et al., 2008; Longe et al., 2009; Murray et al., 2015). Area 11 is differentially connected with more medial and more lateral areas, namely subarea 11m shares more connectivity with area 14, whereas area 11l is more interconnected to areas 12 and 13 (Carmichael and Price, 1996; Price, 2006). Given this intermediate role of area 11, its inclusion in the lesion extent in the earlier report may support the inconsistent findings. However, differences in stimuli (static vs. dynamic) may have also impacted the ecological relevance, missing fine details provided by naturally expressed emotions in the videos and, lastly, the manual analysis of the eye-tracking data was potentially less precise than the analysis used in the present study. Taken together these studies suggest an important impact of stimulus type and measurement of emotional reactivity on the understanding the role of the lOFC area 13 in emotion regulation. Future studies should further investigate the differences in emotional reactivity due to stimulus type in order to disambiguate these conflicting results.

Finally, damage to the OFC13 caused significant increases in pupil diameter to positive social stimuli, again mirroring the deficits reported after damage to the OFC12 and those of our earlier studies on monkeys with area 11/13 aspiration lesions (Goursaud and Bachevalier, 2020). Increased pupil diameter in humans is thought to represent increases in emotional arousal and cognitive effort that may be caused, in part, by disinhibition of the amygdala. The role of area 13 in mediating arousal to social cues is well-supported in the literature, as lesions *via* aspiration surgeries (Izquierdo et al., 2005; Kalin et al., 2007) or neurotoxin injections (Machado and Bachevalier, 2008) result in decreased fear response in social and nonsocial tasks. Though these studies did not measure arousal, *per se*, it is clear that damage including OFC area 13 disrupts normal behavior in highly arousing contexts. Our studies extended these findings by demonstrating that damage to this OFC subfield yields a dysregulated emotional state during the processing of affective cues from a conspecific. This dysregulated arousal in the presence of emotional social stimuli, similar to the findings reported with lesions to OFC area 12 (above) may be due to a failure to assign appropriate emotional value to social cues, driving increased avoidance of key regions of social stimuli, such as the eyes, and perhaps resulting in the disrupted behavioral patterns and altered emotional regulation shown in other reports.

The parallel between the deficits resulting from OFC13 lesions and those following OFC12 lesions (see above), suggests a complementary role of OFC areas 12 and 13 in perception of social signals and emotional modulation. Given that damage to each region produces similar outcomes, the processing of social and emotional cues may rely on the interaction between OFC12 and OFC13 rather than either region in isolation. In a functional neuroimaging study in monkeys, Tsao et al. (2008b) reported the presence of prefrontal patches of activation that responded more strongly to expressive than to neutral faces. The patches of activity responding the strongest to expressive faces were located within the ventral portion of lOFC area 12 close to the border with the lateral orbitofrontal sulcus, but no patches were found in lOFC area 13. In addition, in a meta-analysis of neuroimaging studies in humans, Zald et al. (2014) dissociated activation between the lateral OFC (areas 11, 13) and the medial OFC (areas 14, 25, and 32) and indicated more robust activation of lOFC area 13 during tasks requiring face monitoring and face discrimination than in medial OFC (areas 14, 25 and 32). Thus, a potential functional distinction between lOFC areas 12 and 13 could be one between the overall monitoring/discrimination of faces strongly recruiting lOFC area 13 compared to lOFC area 12, and the processing of emotional content of faces recruiting lOFC area 12 more than area 13. However, the experimental conditions used in the current study were not sufficient to differentiate between these processes. Thus, it is possible that the two sub-regions may serve similar functions in attention and modulation of arousal in the presence of social and emotional cues. Further studies will be required to more specifically characterize the distinctive roles of lOFC areas 12 and 13 in the monitoring of facial cues.

### OFC Area 14 Lesions Do Not Impact the Modulation of Attention or Arousal to Social or Emotional Cues

Lesions to OFC area 14 spared any disruption in attention to stimuli regardless of social content or emotional valence, and also spared any disruption of emotion regulation. Compared to controls, animals with OFC14 lesions did not differ significantly on overall attention to social or nonsocial stimuli regardless of emotional valence. In addition, animals with OFC14 lesions did not differ from controls in any measure of attention to faces and of arousal to social stimuli. Thus, the results suggest that OFC area 14 alone does not seem critical to these processes, which are likely supported by functional activity in other regions. The lack of effects of OFC14 lesions is particularly intriguing because those lesions included small damage to OFC area 13 that appears similar to the small damage reported after the OFC13 lesions themselves. However, it is important to note that, despite similar percent of cell loss in area 13 following OFC14 and OFC13 lesions, the location of the cell loss differed. Cell loss after OFC13 lesions was visible within the superficial cortical layers across the entire anteroposterior extent of area 13 and covered the cortex in between the lateral and medial orbital sulci, whereas unintended cell loss in area 13 after OFC14 lesions was limited to the cortex within the medial wall of the medial orbital sulcus but included all six cortical layers (see Figure 2).

The absence of broad attentional deficits in the processing of socioemotional stimuli are generally consistent with other reports in nonhuman primates (Noonan et al., 2010), indicating limited impacts on decision making and social valuation following OFC area 14 lesions. The current findings support the notion that area 14 alone is not critical for the regulation of emotion, particularly to threatening cues (Rudebeck et al., 2013). Although some studies suggest that OFC14 is important for the processing of positive appetitive outcomes (Noonan et al., 2011, 2012; Hampshire et al., 2012), the present data did not indicate any changes in attention to positive cues in social or nonsocial contexts following OFC14 lesions and thus did not support the notion that area 14 is essential for this ability. Critically, the videos containing positive social cues, as used in the present study, may not be sufficient to evoke the disruptions of emotion regulation reported in studies administering food and juice rewards. Yet, other studies have suggested a role for OFC14 in the modulation of behavior to nonsocial threatening stimuli (Pujara et al., 2019). Incongruence between our data and those reported in this latter study may result from differences in task structure, as we employed a passive viewing task compared to an active approach-avoid task in the Pujara et al. study. In addition, lesions that include area 14 as well as neighboring medial subgenual regions, such as areas 10, 25 and 32, yield deficits in processing of emotion cues in humans (Dal Monte et al., 2013; Wolf et al., 2014). Taken into context with our data, the impairments to emotion processing in these latter studies are likely due to additional damage to medial subgenual areas known to play a critical role in emotion regulation (Ongür and Price, 2000; Alexander et al., 2019a,b).

The lack of impacts of OFC14 lesions are also surprising given the regions connectivity with areas of the anterior cingulate cortex (ACC) implicated in emotional processing, such as areas 32 and 24 (Ongür and Price, 2000). The ACC has been implicated in reward sensitivity (Manohar and Husain, 2016), uncertainty (Cohen et al., 2005), and behavioral disruption (Camille et al., 2011), as well as in social prediction and decision making (Apps et al., 2016). The lack of impact of OFC14 lesions on the regulation of autonomic arousal is also surprising, given the connectivity with regions such as the hypothalamus that are linked to arousal. However, the OFC14 is part of an emotion processing network and intact function in adjacent regions, such as the ACC, may be sufficient to mount an appropriate arousal response to stimuli used in the current study. In addition, studies have shown modulation of autonomic arousal in mOFC to be linked to positive reinforcement outcomes (Nagai et al., 2004), and the stimuli used in the current study may not have been sufficiently reinforcing or engaging to observe alterations in autonomic arousal. Future studies should probe whether OFC14 lesions impact baseline arousal levels (absent of emotional stimuli) and stimuli associated with appetitive outcomes to confirm and extend these findings. Together, our findings suggest that OFC14 alone, despite extensive connections to important regions for social processing and the modulation of arousal, plays a more limited role in emotional processing and that redundant emotional processing within other OFC and medial subgenual regions is sufficient to support emotion regulation when passively viewing dynamic stimuli as in the present study.

## Conclusions and Limitations

Together, the data indicate that areas within the lateral OFC network (areas 12 and 13) are more critically involved in perceptual processing of emotional social cues and modulation of autonomic responses than OFC14. This functional dissociation is in accord with the differences in anatomical connectivity already reported between the lateral and medial OFC networks (Barbas and Pandya, 1989; Barbas, 2000). These findings increase our understanding of the role of face-responsive neurons in OFC areas 12 and 13 during directed attention to salient socioemotional cues. In addition, these findings suggest that one mechanism for directing attention may be through the flexible valuation of social cues, as demonstrated by altered autonomic arousal to positive social stimuli, possibly in concert with regions like the amygdala that are important for emotional arousal. These findings also extend our understanding of the OFC areas 12 and 13 that suggest that fiber-sparing lesions of those areas in isolation are not sufficient to disrupt broader emotional and social processing, given that these lesions spared the preference for viewing social compared to nonsocial scenes and the overall modulation of attention across valence.

Nevertheless these conclusions should be considered in light of the experimental limitations inherent to our study, including small sample sizes with unbalanced sex ratios, response variability in subjects of our control group, variations in lesion extent and unintended damage, cortical reorganization following permanent lesions, mixing of novel and familiar nonsocial stimuli across valence, and limitations with our exploratory pupil analysis lacking critical details. Thus, additional studies with a larger sample size are needed to confirm the present findings and an improved male-to-female gender ratio should be used to investigate potential gender differences in attention to social stimuli that have already been reported in monkeys and humans (Proverbio et al., 2008; Payne and Bachevalier, 2013; Simpson et al., 2016). Future studies may also explore the potential for reversible lesions using DREADDS inactivation to limit the effects of cortical reorganization following permanent lesions. In addition, though all efforts were made to present balanced content across the social and nonsocial emotional conditions, we cannot exclude the possibility that the inconsistent presence of familiar vs. novel stimuli within the nonsocial scenes may have masked important group differences in the analyses of the nonsocial stimuli. However, we believe this limitation to be minor, given equal levels of attention to nonsocial neutral stimuli (all novel) and nonsocial positive stimuli (all familiar) and the low levels of attention produced by nonsocial negative stimuli that included a mix of novel and familiar content. Finally, there were clear limitations on the data collected for pupillary arousal analysis. Although these analyses were included *post hoc*, the corrections we made to the raw data (similar to those reported in Goursaud and Bachevalier, 2020) and the existence of changes in arousal together with the changes in scanning patterns of social cues indicate a value to include these exploratory data in this study.

To sum, despite these limitations, we believe that the data presented here paralleled earlier reports in the literature and capture the critical role of the lateral OFC network in attention to socioemotional cues. Together with the presence of face-selective neurons in the lateral OFC network, the data suggest that the lateral OFC network may set the stage for multidimensional information processing related to face, person, and emotion and may be involved in social judgments (Cicerone and Tanenbaum, 1997; Ligneul et al., 2017).

## Data Availability Statement

The datasets generated for this study are available on request to the corresponding author.

## Ethics Statement

The animal study was reviewed and approved by Institutional Animal Care and Use Committee of Emory University and align with the standards set by the NIH Guide for the Care and Use of Laboratory Animals.

## Author Contributions

LM and JB contributed to the conception and design of the study. LM conducted experimental testing, conducted statistical analysis and wrote the first draft of the manuscript. JB performed surgeries and wrote sections of the manuscript. Both authors contributed to manuscript revision, read and approved the final version.

## Conflict of Interest

The authors declare that the research was conducted in the absence of any commercial or financial relationships that could be construed as a potential conflict of interest.
