# Supplementary material for: Damage to Orbitofrontal Areas 12 and 13, but Not Area 14, Results in Blunted Attention and Arousal to Socioemotional Stimuli in Rhesus Macaques
Source: Front Behav Neurosci. 2020 Sep 8;14:150. doi: 10.3389/fnbeh.2020.00150 (PMC7506161; doi:10.3389/fnbeh.2020.00150)
Supplement: Supplementary file 1 [file Table_1.DOCX]

Supplementary Material

# Supplementary Figure 1.

|  | **Area 12** | | | |
| --- | --- | --- | --- | --- |
|  | L | R | Avg. | W |
| OFC12-1 | 20.17 | 36.99 | 28.58 | 7.46 |
| OFC12-2 | 48.60 | 34.35 | 41.47 | 16.69 |
| OFC12-3 | 14.13 | 17.48 | 15.80 | 2.47 |
| OFC12-4 | 26.82 | 4.27 | 15.54 | 1.15 |
| **X** | **27.43** | **23.27** | **25.35** | **6.94** |
|  | **Area 13** | | | |
|  | L | R | Avg. | W |
| OFC13-1 | 2.74 | 1.95 | 2.34 | 0.05 |
| OFC13-2 | 18.95 | 21.58 | 20.26 | 4.09 |
| OFC13-3 | 14.79 | 0.00 | 7.40 | 0.00 |
| OFC13-4 | 12.67 | 27.59 | 20.13 | 3.49 |
| OFC13-5 | 31.11 | 22.37 | 26.74 | 6.96 |
| **X** | **16.05** | **14.70** | **15.37** | **2.92** |
|  | **Area 14** | | | |
|  | L | R | Avg. | W |
| OFC14-1 | 45.09 | 36.85 | 40.97 | 16.61 |
| OFC14-2 | 48.93 | 32.83 | 40.88 | 16.06 |
| OFC14-3 | 0.08 | 0.38 | 0.23 | 0.00 |
| OFC14-4 | 5.65 | 47.67 | 26.66 | 2.69 |
| **X** | **24.94** | **29.43** | **27.18** | **8.84** |
|  |  |  |  |  |

**Histological lesion extent for orbitofrontal regions.** The table depicts the percent damage to OFC areas 12, 13, and 14 for each treatment group. For each case damage is listed for the left (L) and right (R) hemispheres, and the average (Avg) and weighted average (W) are computed. The weighted average is calculated as W = (L*R)/100. Below each group in bold is the average value for each parameter (X).

# Supplementary Figure 2

| **Subject Group Membership and Identification** | **Sex** | **Average Fixation Duration to the Scene In Seconds** | | | | | |
| --- | --- | --- | --- | --- | --- | --- | --- |
|  |  | **Social Stimuli** | | | **Nonsocial Stimuli** | | |
|  |  | **Negative** | **Neutral** | **Positive** | **Negative** | **Neutral** | **Positive** |
| **Control-10** | F | 0.71 | 0.46 | 0.32 | 0.28 | 0.36 | 0.33 |
| **Control-11** | M | 1.66 | 1.18 | 0.95 | 0.49 | 0.40 | 0.00 |
| **Control-12** | M | 1.98 | 1.51 | 1.37 | 0.23 | 0.60 | 0.10 |
| **Control-13** | M | 1.17 | 0.73 | 0.81 | 0.26 | 0.32 | 0.02 |
| **OFC12-1** | M | 1.25 | 1.29 | 2.15 | 0.16 | 0.49 | 0.11 |
| **OFC12-3** | M | 0.98 | 0.56 | 0.86 | 0.35 | 0.26 | 0.04 |
| **OFC12-2** | M | 1.06 | 0.68 | 0.70 | 0.30 | 0.33 | 0.04 |
| **OFC12-4** | F | 0.95 | 1.19 | 0.63 | 0.28 | 0.31 | 0.00 |
| **OFC13-1** | M | 1.57 | 0.97 | 0.82 | 0.36 | 0.38 | 0.07 |
| **OFC13-2** | F | 1.06 | 1.35 | 0.91 | 0.36 | 0.44 | 0.02 |
| **OFC13-3** | F | 1.19 | 0.54 | 0.94 | 0.45 | 0.32 | 0.03 |
| **OFC13-4** | F | 0.61 | 0.75 | 0.53 | 0.24 | 0.21 | 0.03 |
| **OFC13-5** | F | 1.12 | 1.21 | 1.17 | 0.37 | 0.14 | 0.01 |
| **OFC14-1** | M | 2.16 | 2.21 | 1.50 | 0.30 | 0.37 | 0.06 |
| **OFC14-2** | F | 1.58 | 1.90 | 0.90 | 0.35 | 0.30 | 0.00 |
| **OFC14-3** | M | 1.99 | 1.52 | 1.33 | 0.41 | 0.40 | 0.01 |
| **OFC14-4** | F | 0.78 | 0.58 | 0.53 | 0.30 | 0.12 | 0.02 |

**Supplementary Figure 2A.** The table depicts the average fixation duration to social and nonsocial scenes in seconds. For each subject, values are shown for the negative, neutral and positive valence conditions separately for social and nonsocial scenes.

| **Subject Group Membership and Identification** | **Sex** | **Percent Fixation to Regions of Interest within Social Stimuli** | | | | | | | | |
| --- | --- | --- | --- | --- | --- | --- | --- | --- | --- | --- |
|  |  | **Body** | | | **Eyes** | | | **Mouth** | | |
|  |  | **Negative** | **Neutral** | **Positive** | **Negative** | **Neutral** | **Positive** | **Negative** | **Neutral** | **Positive** |
| **Control-10** | F | 56.45% | 62.62% | 53.49% | 16.85% | 11.01% | 24.92% | 7.45% | 6.29% | 0.00% |
| **Control-11** | M | 85.84% | 85.82% | 83.30% | 28.59% | 44.02% | 51.71% | 6.75% | 5.31% | 15.49% |
| **Control-12** | M | 97.11% | 92.04% | 97.82% | 52.13% | 54.22% | 59.75% | 14.10% | 0.41% | 12.38% |
| **Control-13** | M | 63.43% | 76.48% | 58.94% | 19.45% | 11.35% | 21.22% | 4.53% | 7.79% | 1.86% |
| **OFC12-1** | M | 35.41% | 85.43% | 61.89% | 0.00% | 3.55% | 3.62% | 2.60% | 14.65% | 5.12% |
| **OFC12-3** | M | 70.95% | 69.97% | 43.46% | 16.10% | 9.09% | 19.80% | 3.10% | 0.00% | 4.88% |
| **OFC12-2** | M | 78.78% | 87.50% | 73.41% | 0.00% | 0.00% | 16.05% | 11.11% | 10.05% | 0.00% |
| **OFC12-4** | F | 48.94% | 84.69% | 82.48% | 5.76% | 28.20% | 30.90% | 4.08% | 16.38% | 0.00% |
| **OFC13-1** | M | 54.95% | 88.89% | 33.19% | 2.35% | 23.99% | 16.66% | 5.83% | 0.00% | 5.96% |
| **OFC13-2** | F | 79.70% | 78.59% | 60.99% | 9.69% | 37.38% | 17.21% | 12.17% | 1.12% | 9.52% |
| **OFC13-3** | F | 67.31% | 69.66% | 51.92% | 15.07% | 18.07% | 30.64% | 10.25% | 0.00% | 0.00% |
| **OFC13-4** | F | 59.08% | 78.78% | 50.12% | 9.62% | 20.97% | 9.63% | 2.04% | 6.85% | 3.49% |
| **OFC13-5** | F | 58.39% | 77.30% | 85.68% | 8.58% | 0.92% | 28.87% | 7.28% | 4.36% | 20.77% |
| **OFC14-1** | M | 75.93% | 79.31% | 66.49% | 24.92% | 35.49% | 58.07% | 5.13% | 0.93% | 1.01% |
| **OFC14-2** | F | 60.54% | 79.15% | 71.66% | 7.99% | 29.10% | 31.30% | 8.47% | 6.93% | 15.10% |
| **OFC14-3** | M | 75.03% | 88.75% | 90.88% | 22.62% | 44.28% | 60.42% | 6.94% | 3.07% | 11.32% |
| **OFC14-4** | F | 60.41% | 81.61% | 78.44% | 6.11% | 32.79% | 26.11% | 12.93% | 1.36% | 8.53% |

**Supplementary Figure 2B.** The table depicts the average percent fixation duration to key regions of social stimuli. For each subject, values are shown for the negative, neutral and positive valence conditions separately for the body, eye, and mouth regions.

| **Subject Group Membership and Identification** | **Sex** | **Average Pupil Diameter in Pixels** | | | | | |
| --- | --- | --- | --- | --- | --- | --- | --- |
|  |  | **Social Stimuli** | | | **Nonsocial Stimuli** | | |
|  |  | **Negative** | **Neutral** | **Positive** | **Negative** | **Neutral** | **Positive** |
| **Control-10** | F | 38.14 | 56.61 | 12.44 | 50.22 | 42.89 | 45.50 |
| **Control-11** | M | 23.20 | 48.90 | 21.29 | 41.89 | 39.67 | 46.00 |
| **Control-12** | M | 27.12 | 39.80 | 23.57 | 39.67 | 36.17 | 33.47 |
| **Control-13** | M | 34.93 | 47.88 | 19.86 | 37.08 | 35.67 | 36.83 |
| **OFC12-1** | M | 17.07 | 41.20 | 25.93 | 35.55 | 34.74 | 38.13 |
| **OFC12-3** | M | 27.80 | 39.53 | 34.17 | 37.88 | 39.44 | 31.28 |
| **OFC12-2** | M | 12.67 | 39.75 | 33.57 | 39.06 | 34.27 | 35.86 |
| **OFC12-4** | F | 36.47 | 56.18 | 34.14 | 52.06 | 45.29 | 48.92 |
| **OFC13-1** | M | 41.26 | 52.95 | 27.22 | 48.93 | 44.58 | 44.90 |
| **OFC13-2** | F | 27.73 | 50.41 | 24.57 | 44.53 | 41.91 | 38.29 |
| **OFC13-3** | F | 34.63 | 40.59 | 30.40 | 40.67 | 39.28 | 38.73 |
| **OFC13-4** | F | 27.89 | 50.09 | 28.44 | 34.61 | 32.44 | 32.93 |
| **OFC13-5** | F | 33.94 | 42.50 | 30.71 | 37.52 | 38.33 | 35.28 |
| **OFC14-1** | M | 22.70 | 39.35 | 22.65 | 37.42 | 37.81 | 37.25 |
| **OFC14-2** | F | 30.93 | 42.81 | 19.26 | 35.50 | 33.61 | 39.25 |
| **OFC14-3** | M | 27.50 | 42.46 | 25.07 | 38.94 | 36.44 | 33.48 |
| **OFC14-4** | F | 30.29 | 50.95 | 20.70 | 42.77 | 44.08 | 40.36 |

**Supplementary Figure 2C.** The table depicts the average pupil diameter observed for social and nonsocial scenes in pixels. For each subject, values are shown for the negative, neutral and positive valence conditions separately for social and nonsocial scenes.
